# DNA lesion bypass and the stochastic dynamics of transcription-coupled repair

**DOI:** 10.1073/pnas.2403871121

**Published:** 2024-05-08

**Authors:** Michael D. Nicholson, Craig J. Anderson, Duncan T. Odom, Sarah J. Aitken, Martin S. Taylor

**Affiliations:** ^a^Cancer Research United Kingdom Scotland Centre, Institute of Genetics and Cancer, University of Edinburgh, Edinburgh EH4 2XU, United Kingdom; ^b^Medical Research Council Human Genetics Unit, Institute of Genetics and Cancer, University of Edinburgh, Edinburgh EH4 2XU, United Kingdom; ^c^Division of Regulatory Genomics and Cancer Evolution (B270), German Cancer Research Center, Heidelberg 69120, Germany; ^d^Cancer Research United Kingdom Cambridge Institute, University of Cambridge, Cambridge CB2 0RE, United Kingdom; ^e^Medical Research Council Toxicology Unit, University of Cambridge, Cambridge CB2 1QR, United Kingdom; ^f^Department of Histopathology, Cambridge University Hospitals National Health Service Foundation Trust, Cambridge CB2 0QQ, United Kingdom

**Keywords:** mutation, damage, transcription, repair, mathematical modeling

## Abstract

DNA base damage is a major source of oncogenic mutations and disruption to gene expression. The stalling of RNA polymerase II (RNAP) at sites of DNA damage and the subsequent triggering of repair processes have major roles in shaping the genome-wide distribution of mutations, clearing barriers to transcription, and minimizing the production of miscoded gene products. Despite its importance for genetic integrity, key mechanistic features of this transcription-coupled repair (TCR) process are controversial or unknown. Here, we exploited a well-powered in vivo mammalian model system to explore the mechanistic properties and parameters of TCR for alkylation damage at fine spatial resolution and with discrimination of the damaged DNA strand. For rigorous interpretation, a generalizable mathematical model of DNA damage and TCR was developed. Fitting experimental data to the model and simulation revealed that RNA polymerases frequently bypass lesions without triggering repair, indicating that small alkylation adducts are unlikely to be an efficient barrier to gene expression. Following a burst of damage, the efficiency of transcription-coupled repair gradually decays through gene bodies with implications for the occurrence and accurate inference of driver mutations in cancer. The reinitation of transcription from the repair site is not a general feature of transcription-coupled repair, and the observed data is consistent with reinitiation never taking place. Collectively, these results reveal how the directional but stochastic activity of TCR shapes the distribution of mutations following DNA damage.

## Significance

Damage to DNA can interfere with crucial cellular processes such as the transcription of genes into RNA and can ultimately lead to mutations, DNA sequence changes, that are inherited by subsequent generations of cells and organisms. Transcription-coupled repair (TCR) works to remove damage from genes that are being used by a cell. We reveal mechanistic details of how TCR works, its efficiency, and how that changes through the length of a gene. This helps understand how cells deal with a burst of DNA damage, for example, from sunburn or chemotherapeutic treatment, and where the resulting genetic damage is likely to occur, with implications for cancer risk and treatment.

**Fig. 1.**
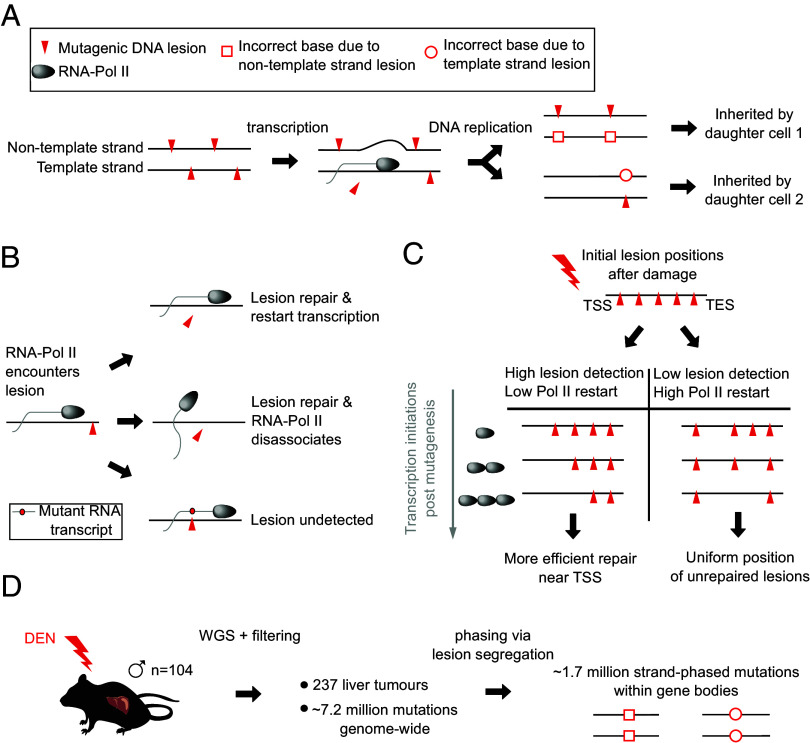
Quantifying the dynamics of transcription-coupled DNA repair with lesion-strand phased mutations and gene expression measures. (*A*) Template strand DNA damage is alleviated during transcription by transcription-coupled repair. Lesions that persist to replication can cause heritable mutations created through incorrect base-pairing. (*B*) Alternate possible outcomes from transcription over a lesion-containing template DNA strand. (*C*) Schematic of lesion clearance due to TCR following damage. The pattern of remaining lesions as a function of both expression and genic position is dependent on the sensitivity of RNAP and whether the RNAP restarts following repair. (*D*) We utilize strand-phased mutation data from 237 liver tumors induced by exposing male C3H mice to a single dose of DEN.

Accurate and efficient DNA replication and DNA transcription are essential for life. However, cellular DNA is continuously assaulted with damage arising from both endogenous and exogenous sources. With hundreds of thousands of DNA adducts forming per genome per day, crucial molecular processes can be severely inhibited (1). Damage falling within transcribed regions poses particularly acute challenges, potentially interfering with accurate and efficient transcription, as well as risking the formation of heritable, protein-altering mutations. Transcription-coupled repair (TCR), a highly conserved branch of the nucleotide excision repair pathway (2, 3), assists in minimizing the risk of such aberrant outcomes (Fig. 1*A*). Triggered by the stalling of actively transcribing RNA polymerase II (RNAP), TCR excises the stalling lesion and, by using the nontranscribed strand as a template for synthesis, results in repaired, lesion-free DNA.

Frequent RNAP stalling potentiates dysregulation of homeostatic expression and increased transcription–replication complex collisions (4). On the other hand, uncleared damage risks transcriptional mutagenesis (5) and incorrect base pairing at replication. Thus, a balance between damage tolerance and clearance must be struck. Central to understanding this balance, and our ability to quantitatively map damage to cellular outcome, is the measurement of how the transcriptional machinery interacts with damage. In this study, we focus on two key elements of this interaction: the sensitivity with which RNAPs detect damage and trigger TCR and how frequently RNAPs reinitiate transcription following repair (Fig. 1*B*).

The efficiency of TCR initiation is expected to be influenced by lesion type (4, 6). Smaller adducts, such as the oxidative stress–induced 8-oxoguanine, are bypassed with relative ease by RNAP (7), while more bulky, helix-distorting lesions, e.g., UV-induced pyrimidine-dimers, provide a more stringent roadblock to transcribing RNAP, which may only rarely be bypassed (8, 9). When RNAP stalling and repair do occur, transcription must be rapidly resumed to maintain cellular function. It was commonly thought that stalled RNAPs resumed transcription from the damaged site (10); however, recent work has demonstrated disassociation of RNAP following TCR at UV-induced pyrimidine dimers (11). Without RNAP restart, further RNAP transcription initiations at a given gene’s promoter are required, potentially necessitating numerous transcription initiations to clear a gene body of multiple lesions and to generate a complete RNA transcript. While the bypass efficiency for varied lesions can be quantified in vitro (12), an integrative picture summarizing the outcomes of transcriptional machinery encountering adducts in vivo is lacking.

For TCR-inducing lesions, we reasoned that analyzing mutation burden as a function of both gene expression and genic position would provide insight into TCR mechanics. DNA damage that avoids repair and persists to replication can result in incorrect base-pairing, thus generating heritable mutations that are detectable in the damaged cell’s progeny. Supposing that template strand lesions consistently stall RNAP, triggering lesion excision and repair and subsequent RNAP disassociation, then any downstream lesions will require a second RNAP for detection and clearance. Under this model, the 5′ end of moderately expressed genes would be cleared of lesions, but the 3′ end would remain unrepaired (Fig. 1*C*). If this positional bias in lesions persists through to DNA replication, then a sigmoidal mutational pattern through the gene bodies would be expected, with the curve progressively moving toward the 3′ end as transcription increases. Alternatively, if RNAPs consistently reinitiate transcription following lesion detection and repair, then no positional bias in lesion clearance should be expected, and hence, a more uniform mutation burden through the gene body is predicted (Fig. 1*C*). Therefore, observing mutational patterns caused by template strand lesions as a function of genomic position and gene expression potentially offers a window into the mechanics of TCR.

As RNAP is only expected to trigger the repair of damage on the transcriptional template strand, a prerequisite for using mutation patterns to accurately infer the activity of TCR is the ability to resolve the lesion containing strand. Prior studies (13, 14) have relied on inferences from the biochemistry of mutagenesis for lesion strand resolution, for example, assuming that C-> T mutations from UV photoadducts involve the C nucleotide rather than the G of the complementary strand. Such inferences can be confounded by atypical adducts (15), and the spectrum of adducts produced by other mutagens is generally less well understood. An alternative strategy is to ab initio phase the strand of DNA damage. Following a burst of mutagenic damage in a single cell cycle, most mutations arise through replication using a damaged base as a template (16). Through the semiconservative replication of DNA, the two complementary strands of a DNA duplex will template the new synthesis of two sister chromatids that, through mitosis, segregate into separate daughter cells (Fig. 1*A*). Each daughter cell lineage receives the DNA lesions, and ultimately mutations, from just one of the parental DNA strands. This DNA lesion segregation (16) results in chromosome scale, strand asymmetric mutation patterns that can be used to confidently discriminate the DNA lesion strand (16). Through comparison to gene annotation, the DNA lesion strand can be further resolved as either the transcriptional template or nontemplate strand [Fig. 1*A*; (17)].

To explore the mechanism and efficiency of TCR in vivo, with spatial precision and lesion strand resolution, we have exploited an established mouse model of diethylnitrosamine (DEN)-induced liver cancer (18, 19) (Fig. 1*D*). DEN is bioactivated into a potent but short-lived mutagen by the hepatocyte expressed enzyme Cyp2e1. This generates a range of DNA alkylation adducts, including the principal mutagenic lesion O^4^-ethyldeoxythymidine (18). Tumors reliably develop within 24 wk of a single acute exposure to DEN; each of these represents a clonal expansion of one post-mutagenesis cell whose genome typically contains 60,000 base substitution mutations, and exhibits the pronounced mutation asymmetry of lesion segregation (16).

Here, we examine strand-phased mutational patterns as a function of gene expression and lesion position to quantify the mechanics of TCR. We present a probabilistic mathematical model, incorporating the key mechanistic features of the TCR process, which is able to recapitulate the mutation patterns of DEN-induced tumor genomes. Analyzing the murine liver data through the mathematical model, we show that for alkylation DNA adducts such as those created via DEN exposure, the initiation of TCR is stochastic, with frequent transcription occurring over mutagenic lesions. Overall, our modeling approach provides a framework for translating strand-phased mutation data to the mechanics of TCR.

## Results

### TCR Shapes Mutation Patterns through the Gene Body in DEN-Induced Tumor Genomes.

We aimed to identify the speculated mutational patterns in the genomes of DEN-induced murine liver tumors. As previously described (16), using lesion segregation, we were able to call approximately 1.7 million high-confidence, strand-resolved mutations within transcribed regions from 237 tumor genomes. Matching gene expression measures were generated contemporaneously by total cellular RNA sequencing on healthy liver tissue from untreated litter-mates (16), and nascent transcription rates estimated from intron mapping reads (17).

**Fig. 2.**
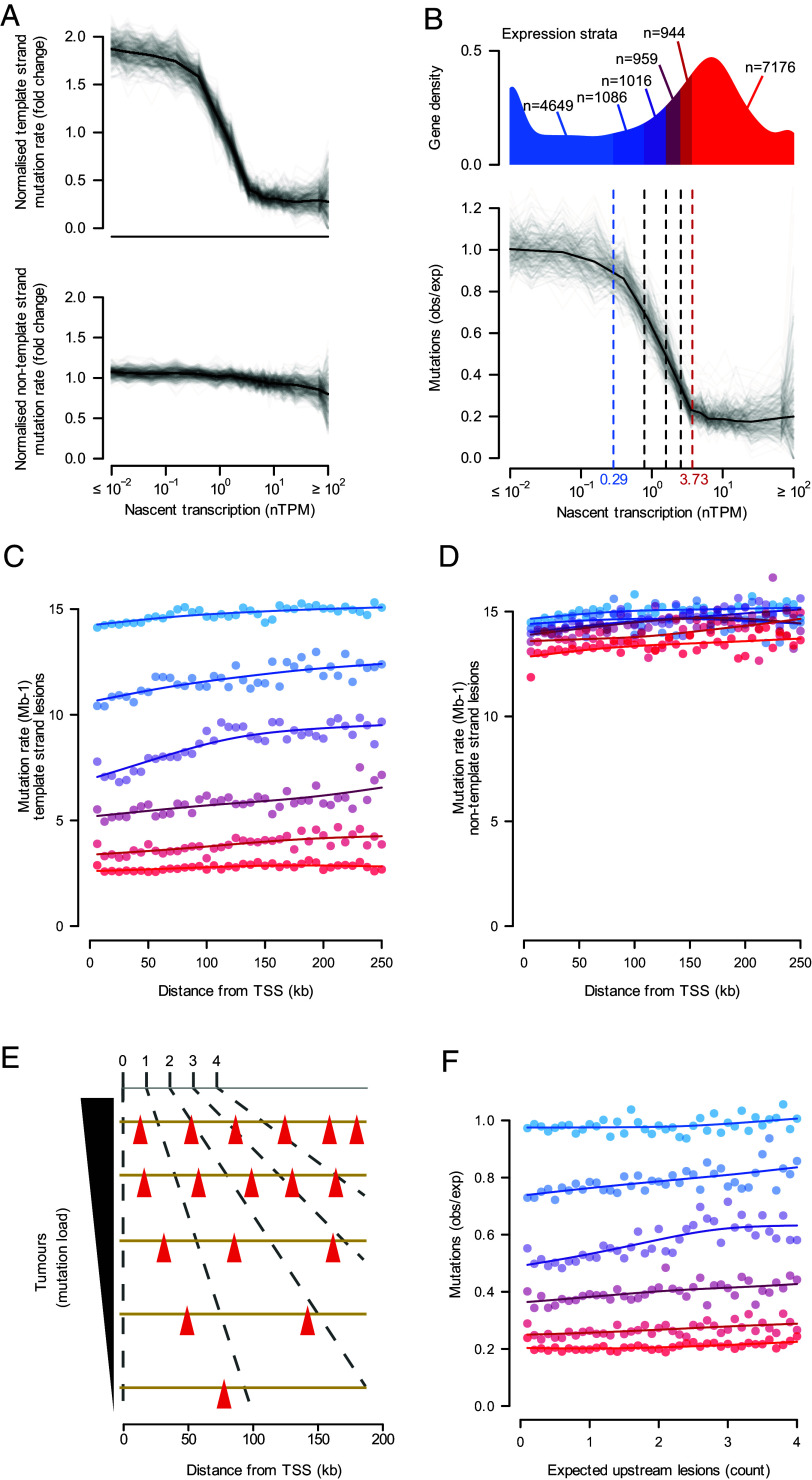
Transcription-coupled repair shapes the distribution of mutations through the body of expressed genes. (*A*) Tumors (gray curves) consistently show the same normalized profile of transcription-coupled repair: increased expression (x-axis; plotted on the log scale) corresponding to reduced mutation rate (y-axis) for lesions on the transcription template strand (*Upper*). The mutation rate per tumor is normalized to the average for all genes in the tumor. For lesions on the nontemplate strand (*Lower*), increased expression only subtly influences normalized mutation rate. The black line is the median of the per-tumor rates. (*B*) The *Lower* panel shows observed versus expected mutations (y-axis) calculated as the ratio of template strand mutation rate to nontemplate strand mutation rate plotted against nascent transcription rate per tumor (x-axis). Expression >3.73 nascent transcripts per million (nTPM) does not further decrease the mutation rate. In subsequent analyses, gene expression is binned into six strata of nascent gene expression (*Upper*) blue→red denotes increasing expression, dashed lines demarcating strata boundaries (*Methods*). (*C*) Mutation rates for genes with template strand lesions. Genes classified by expression strata and mutation rates calculated in 5 kb consecutive windows from the transcription start site (TSS). Points show observed data and curves show best-fit splines (3 degrees of freedom). (*D*) As for (*C*) but considering genes with nontemplate strand lesions. (*E*) Schematic of per-tumor normalization to calculate the number of expected upstream lesions (red triangles) for each analysis window (*Methods*). (*F*) Observed versus expected mutations (y-axis) calculated as the ratio of template to nontemplate strand. Expected upstream lesion count (x-axis) categories as per (*E*). Points represent data while curves show best-fit splines (3 degrees of freedom). Genes with intermediate levels of expression (strata 2 to 5) exhibit a lower mutation rate at their 5′ end.

We first assessed the relationship between strand-specific mutation burden and gene expression. Consistent with TCR playing a dominant role in DEN-induced lesion repair, the mutation rate due to template strand lesions (hereafter, template mutation rate) markedly decreased with increasing transcription (Fig. 2*A*). We also observed that the mutation rate due to nontemplate strands lesions (hereafter, nontemplate mutation rate) was modestly reduced (Fig. 2*A*), which may occur due to greater chromatin accessibility in highly expressed genes (17).

To isolate the signal of only TCR, we use the nontemplate mutation rate as the *expected* mutation rate (TCR absent) and compare with the *observed* mutation rate (TCR present) on the template strand. The observed:expected mutation rate quantifies the reduction in mutation burden due to template strand repair; observed:expected values of 1 imply equal lesion burden on both the template and nontemplate strand at DNA replication, suggesting a lack of TCR. In contrast an observed:expected value of 0 implies the complete removal of template strand lesions. This resulted in dose–response type patterns in each of the 237 tumor genomes (Fig. 2*B*). Mutation rates from different tumors may be expected to depend on the state of the tumor’s ancestral cell at mutagenesis, for example, the cell cycle phase at DEN exposure. However, by fitting log–logistic functions (20)—commonly used to quantify dose–response relationships—the shape of the mutation rate decay was found to be remarkably homogeneous (*SI Appendix*, *Extended Data* Fig. S1 *A* and *B*). As described previously (17) at high transcription levels the mutation rate plateaued, suggesting that the remaining mutagenic lesions were largely invisible to TCR. Invisible lesions potentially reflect subsets of lesions that are less efficient at stalling RNAPs or lesions in less recognizable genomic contexts; prior analysis of these data supports that lesions in certain trinucleotide contexts are less permissive to repair (17). Given the consistency of the TCR pattern over individual genomes, henceforth, we analyzed the aggregated data across all genomes.

In order to jointly examine the effect of both expression and the genic position of lesions, the gene expression distribution was binned into six expression strata (Fig. 2 *B*, *Top* panel; *SI Appendix*, *Extended Data* Fig. S1*C*). Strata boundaries were chosen to balance accurately reflecting the variation over expression and to diminish noise by ensuring a sufficient number of genes per stratum. For each stratum, we measured the mutation rate aggregated over all genes in that stratum in consecutive 5 kb windows from the transcription start site (TSS). This demonstrated subtly (approximately 3.5%) lower mutation rates for both template and nontemplate strand lesions at the 5′ end of nonexpressed genes (Fig. 2*C*). This trend was also seen for the nontemplate strand at all expression strata (Fig. 2*D*).

We extended our analyses of observed:expected mutation rates (defined above) to focus on positional biases in mutation burden specifically due to TCR, negating potential confounding factors such as 5′ end effects and enhanced non-TCR surveillance. We also recognized that as transcription is a processive and directional process, the probability of an upstream lesion on the same template strand could influence the TCR efficiency at a given gene position. Consequently, both the upstream sequence composition and per tumor burden of lesions (inferred from mutations) could influence the repair efficiency of a focal analysis window. Addressing these concerns, we created a normalized gene-position measure based on the expected number of upstream lesions that was calculated for each analysis window of each gene, in each tumor, prior to aggregated analysis (*Methods*) (Fig. 2*E*).

Comparison of the observed:expected mutation rates to the expected upstream lesion number (Fig. 2*F* and *SI Appendix*, *Extended Data* Fig. S1 *D*–*K*) leads to several immediate conclusions. First, the observed:expected mutation rate is approximately 1 for the lowest expressed genes (stratum 1), which indicates that, as expected, there is no TCR in the absence of detected transcription. Second, for intermediately expressed genes (strata 2 to 5) we see a linear increase in the mutation rate through the gene body—consistently found when considering only short, or only long genes (*SI Appendix*, *Extended Data* Fig. S1 *I* and *J*); suggesting that TCR efficiency decays approximately linearly with the upstream lesion number. Finally, the highly expressed genes, with >10 nascent transcripts per millions (nTPM), show negligible decay in TCR efficiency through the gene body, indicating that all detectable lesions have been removed. By comparing the observed linear decay in TCR efficiency through gene bodies to the hypothetical mutation pattern scenarios (Fig. 1*C*), these data support a model in which RNAP repairs 5′ lesions before downstream 3′ lesions, with regular disassociation of RNAP following repair. To robustly quantify the mechanistic origins of these effects we developed a mathematical model of TCR.

### Mathematical Model for Transcription-Coupled Repair Dynamics.

**Fig. 3.**
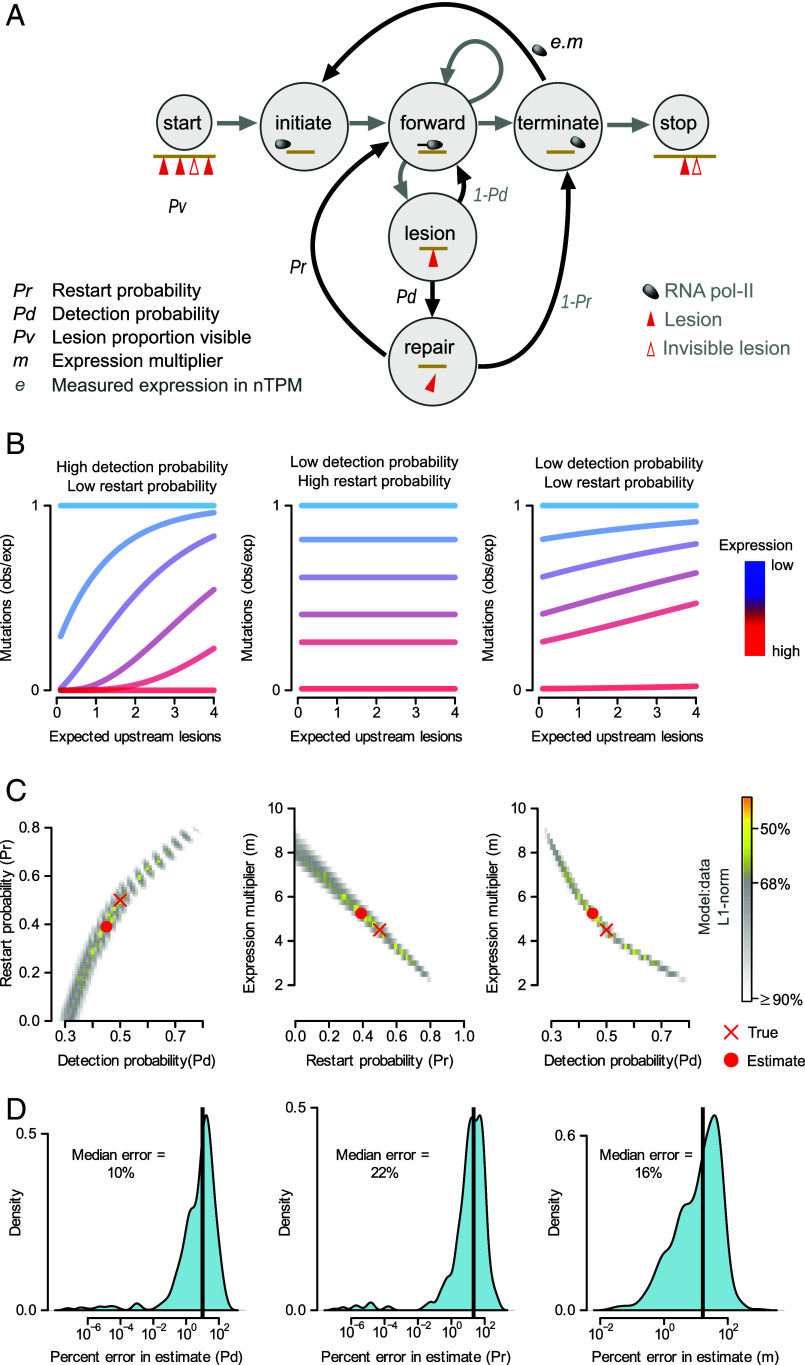
Mathematical model of transcription-coupled repair dynamics. (*A*) Mathematical model of TCR dynamics. A string of nucleotides (yellow line) with DNA lesions (red triangles) is subject to transcription (gray arrows) and probabilistic TCR events (black arrows). On encountering a lesion, the probability of its detection (*Pd*) and of polymerase restart following lesion repair (*Pr*) are independent model variables. The fraction of lesions visible to TCR (*Pv*) and an expression multiplier parameter (*m*) are additional independent variables. (*B*) Example mutation rate profiles generated analytically by the model under varied qualitative parameter regimes. From left to right, the numerical parameters of (*Pd, Pr, m, Pv*) used were: (1,0.25,1.5,1); (0.25,1,1.5,1); (0.25,0.25,1.5,1). Expression level of gene sets denoted by color with red to blue representing high to low expression, respectively (as per Fig. 2*B*). (*C*) An analytic inference scheme was developed to infer model parameters. The heat map of the Manhattan distance between obs:exp_theory_ to simulated data is shown. Shading is determined by whether the obs:exp_theory_ to simulation distance is smaller than the distance between bootstrapped simulated data and the original simulated data, at the displayed quantile levels. Yellow shading concentrated around true parameters illustrates that while errors in estimates are correlated, the true parameters are identifiable. (*D*) Across a wide range of simulated datasets, true parameters can be recovered with small errors. The vertical black line denotes median percentage error.

We defined a Markov chain model (Fig. 3*A*) characterizing the dynamics of transcribing RNAPs in the interim period between DNA damage and replication. To model the initial damage distribution, we selected random positions through gene bodies. Following damage RNAPs sequentially initiate transcription and, upon encountering a lesion, the lesion is detected and repaired with probability *Pd.* Following repair, the RNAPs reinitiate transcription at the site of the damage with restart probability *Pr,* else they disassociate from the strand. Since the efficiency of repair appears to saturate at high levels of transcription without complete lesion removal (Fig. 2*B*), we assumed two types of lesions exist: lesions that are visible to TCR and so can be detected with probability *Pd* and TCR-invisible lesions which will not be detected. As mentioned above, TCR-invisible lesions could have altered biochemistry or lie in less recognizable genomic contexts (17); agnostic to mechanism, we include a parameter *Pv* in the mathematical model for the proportion of lesions that are visible.

To match the experimental analysis we consider six expression strata in the model such that the *k*th strata has an associated average expression level, *e_k_*, measured in units of nascent transcripts per million (nTPM). We fixed the numerical values of (*e*_1_, …, *e*_6_) as the median nTPM for each stratum in the experimentally defined expression data. For genes in a given stratum, we assumed that an average of *n_k_* RNAPs initiated transcription between damage and replication. To relate the RNAP initiations in the model to the RNA sequencing measures, we included an expression multiplication factor (*m*) and specify that *n_k_* = *m∗e_k_.* As the per-strata expression values are fixed, the number of RNAP initiations per gene is controlled only through their associated stratum and *m*. Under mild assumptions, such as each produced RNA transcript having equal chance of being sampled in the RNA sequencing, *m* has the further interpretation as the total number of RNA transcription initiations between damage and replication, in units of transcription initiations (×10^6^) (*Methods*).

Using techniques from Markov process theory (*SI Appendix*, *Supplementary File 1*), we numerically determined the mathematical expectation of the template strand lesion count in the model, as a function of genic position and the expression multiplier, *m*. The coding strand lesion burden is obtained by suppressing transcription in the model. Dividing the modeled template lesion count by the coding lesion count gives the proportion of unrepaired lesions obs:exp_theory_, which is directly analogous to the experimentally measured observed:expected mutation rates. Matching the hypothesized lesion patterns (Fig. 1*C*), if RNAPs always restart following repair (*Pr* = 1), then obs:exp_theory_ is constant over gene position (Fig. 3*B*). With no RNAP restart and high RNAP sensitivity, obs:exp_theory_ adopts a sigmoidal shape; while linear gradients emerge for low to medium values of RNAP sensitivity, similar to the experimental observed:expected mutation rates (Fig. 2*F*).

To examine the utility of the model to infer the mechanistic parameters of TCR, DNA damage followed by TCR was simulated at scales mimicking the murine liver data (*Methods*). A wide grid of parameter values was used, with *Pd* and *Pr* ranging between 0 and 1, while the expression multiplier *m* was constrained within a literature-informed plausible regime. As ~20% of lesions remain unrepaired even in highly expressed genes (Fig. 2*F*), we fixed the proportion of TCR-visible lesions, *Pv*, to be 0.8. For a given parameter combination, damage and repair was simulated for ~1.95 million genes (*Methods*), with genes stratified into 6 expression strata as in the experimental data. Each expression strata was associated with the same nascent expression values *e_k_* measured for the murine liver. Thus, for a given *m* and a gene in strata *k*, an average of *m*∗*e_k_* transcription initiations occurred per gene. For a given parameter combination, we aggregated over all simulated genes to construct the simulated observed:expected mutation rates as a function of expected upstream lesions (Fig. 3*B*). The Manhattan distance between the simulated data and the analytically determined obs:exp_theory_ was minimized to estimate the underlying parameters (Fig. 3*C*).

Intuitively, certain parameter combinations could be challenging to uniquely identify, for example, the same amount of damage may be cleared by many polymerases with low detection sensitivity, or a few polymerases with high lesion detection rates. Indeed, correlations in parameter estimates were observed in two-dimensional heat maps illustrating plausible parameter fits (Fig. 3*C*), defined as those parameters such that the distance from obs:exp_theory_ to the simulated data is less than the distance between the original data and bootstrapped original data. For example, overestimation of detection sensitivity often co-occurred with an underestimate of the expression multiplier. Despite this, as model outputs were required to match simulated data over both spatial (position in gene body) and transcriptomic (expression strata) dimensions, we broadly found the true parameters were identifiable in simulated data, with median percent errors of 10%, 22%, and 16% when estimating *Pd*, *Pr*, and *m*, respectively (Fig. 3*D*).

The results above indicate that we can accurately infer model parameters. However, the expression strata thresholds used for the simulated datasets were the same as those that were constructed to be highly informative on the experimental murine data. As a result, the inference accuracy was dependent on the expression multiplier *m*, with an eightfold increase in the median percent error for *Pd* inference between *m* = 0.5 and *m* = 8.5. Consequently, our simulation work likely underestimates the true accuracy of the inference workflow.

### TCR Is Stochastic, and RNAP Frequently Does Not Restart.

**Fig. 4.**
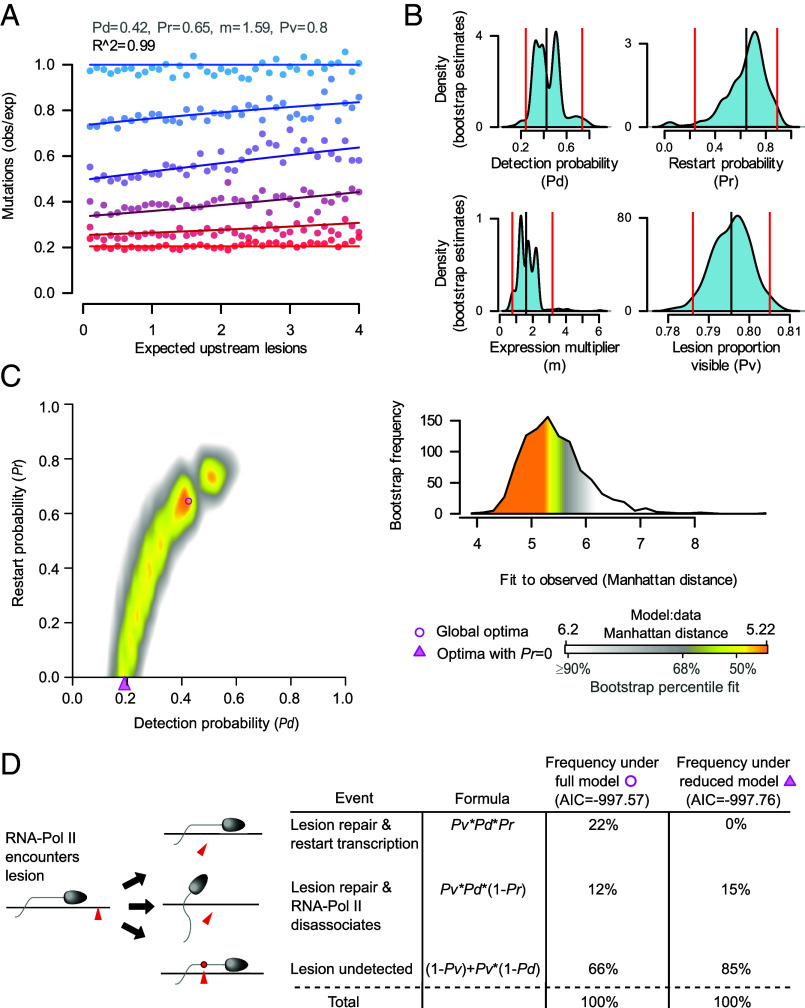
Stochastic dynamics of transcription-coupled repair (TCR) in murine liver tumor genomes. (*A*) Best fit between mathematical model (lines, model parameters in gray text) and data from murine liver genomes (points). Blue→red denotes increasing expression strata (as per Fig. 2*B*). (*B*) Density of parameter estimates obtained from fitting the mathematical model to 1,000 bootstrap samples of mutation data. Red dashed lines indicate bootstrap CIs, black vertical line denotes the estimate from original murine data. (*C*) Heat map (*Left*) showing optimal fits for all grid-search tested values of *Pd* and *Pr* (8.4 × 10^8^ parameter combinations tested). Optimal fits (pink shapes; circle *Pr* ≥ 0, triangle *Pr* = 0) identified from gradient descent exploration initialized by high-quality grid-search fits. Landscape shading from the quantile distribution of fits between the observed data and bootstrap samples of it (*Right*). (*D*) Schematic summary of point estimates of interactions between RNAP and DNA lesions, for the full mathematical model including RNAP restart, and the reduced model without restart. Parameters values for the full model given as optimal in (*A*), and for the reduced model as given in *SI Appendix*, *Extended Data* Fig. S2C.

We analyzed the DEN-induced murine liver tumor mutation data using our mathematical model of TCR, fitting the data as described for the simulations. Despite its simplicity, the model is able to capture the key features of the experimental data (*R^2^* = 0.99), including linear decays in the efficiency of TCR for intermediate expression levels (Fig. 4*A*). For lesions visible to TCR, the lesion detection sensitivity, *Pd*, was estimated to be 0.42, with the 95% CI (CI95) as 0.24 to 0.74 (Fig. 4 *B* and *C*). As the proportion of visible lesions, *Pv*, was estimated to be 0.8 (CI95: 0.79, 0.81), we infer that RNAP frequently transcribes over damage, failing to stall and trigger repair in 66% of lesion encounters (Fig. 4*D*).

The principal mutagenic adduct from DEN exposure is thought to be O^4^-ethyldeoxythymidine (O^4^-EtdT) (18) and the relative bypass efficiency of O^4^-EtdT by mammalian RNAP in vitro is ~60% (21), in close agreement with our inference from in vivo data. For those lesions accessible to TCR, our estimate suggests that each lesion will be transcribed over ~1.5 times before stalling an RNAP and initiating TCR. Transcription over template strand O^4^-EtdT by mammalian Pol II misincorporates ribonucleotides in RNA at a rate of ~50% (21), suggesting widespread transcriptional mutagenesis occurred post-damage in the murine experiments.

The expression multiplier *m* was estimated as 1.59 (CI95: 0.79, 3.18), implying that in the mouse liver cells exposed to DEN, 1.59 million RNAPs initiated transcription between damage and replication. For highly expressed (stratum 6) genes with median expression of 11.15 nTPM, ~18 polymerases are expected to initiate transcription. To assess the validity of this inference, an orthogonal estimate of *m* was determined using estimates of transcription parameters obtained through analysis of single-molecule fluorescence in situ hybridization imaging (*Methods*). Briefly, Bahar Halpern et al. (22) measured the transcription rate and proportion of promoters actively transcribing for seven genes, for which nascent RNA sequencing estimates (*e*) are available in the murine liver experimental data. Combining these values with literature estimates of the time between damage and replication provides estimates of the transcript number produced for each gene (*n*) (*SI Appendix*, *Extended Data* Fig. S2*A*). By the relation *n* = *m*∗*e*, this suggests 2.77 million RNAP initiations occur between damage and replication. As plausible bounds for *m* range over nearly two orders of magnitude (*SI Appendix*, *Extended Data* Fig. S2*B*) (*Methods*), the concordance between the orthogonal estimate to our inferred estimate of 1.59 confirms the robustness of our analytical approach despite the simplifications made.

RNAPs were estimated to restart transcription after 65% (CI95: 24%, 89%) of repair events. As the 95% CI excludes 100%, the null hypothesis that RNAP always restarts from the damaged site after repair is not consistent with these data. Further, parameter combinations that include *Pr* = *0*, denoting the complete absence of polymerase restart, are within the plausible regions as defined above for simulations (Fig. 4*C*). A reduced model without RNAP restart (Pr = 0) provided a near identical fit of the data (*SI Appendix*, *Extended Data* Fig. S2*C*), and model selection analysis indicated that the model without RNAP restart is marginally preferred [Akaike information criterion (AIC) with restart = −997.57, AIC without restart = −997.76]. In the model without restart, lesion detection sensitivity is estimated as 0.19 (CI95: 0.11, 0.25), compared to that of 0.42 for the alternative model. Given that consistent RNAP restart is incompatible with the data, we conclude that transcription restart from the site of stalling is not an obligate feature of TCR. Application of Occam’s razor favors the conclusion that RNAP restart is not a feature of TCR, although the present data do not allow us to exclude the possibility that restart occurs following some TCR events.

## Discussion

In this study, we quantified the interactions between DNA damage and RNAP following exposure of murine hepatocytes to an alkylating agent (DEN) in vivo. DNA lesions that persist to replication are the templates for mutational changes inherited by daughter lineages, which are clonally expanded during tumorigenesis. The resulting mutational readout provides an integrated picture of the repair processes that occur between damage and replication; this offers a complementary approach to the measurements of repair maps, which provide snapshots of repair at specific timepoints (23, 24). By combining strand-phased whole genome sequencing data from 237 mouse liver tumors with RNA sequencing, we showed that transcription-coupled repair leaves a highly reproducible and mechanistically informative footprint when comparing mutation burden to both gene expression and mutation position. To translate the mutation patterns into quantitative estimates of the mechanisms of TCR, we developed a mathematical model of damage and repair able to recapitulate the key features of the data. By analyzing the mouse data through our model we demonstrated that i) lesion bypass of small alkyl adducts is a common feature of transcription, and ii) when lesions do stall RNAPs and elicit TCR, it is common for transcription not to restart from that damaged site (Fig. 4*D*).

Our finding that RNAP frequently bypasses DEN-induced lesions in vivo, extends previous in vitro studies (21, 25) that have considered RNAP bypass of O^4^-EtdT, the principle mutagenic adduct of DEN, and complements findings for other nonbulky adducts (6, 12). However, the exact molecular mechanisms that lead to lesion bypass versus stalling and repair are presently unclear. For alkyl adducts, both nucleotide insertion and RNAP extension past damage can cause prolonged pausing, potentially facilitating damage recognition (25). Thus, contributing factors to the stochasticity of TCR upon lesion encounter may include the sequence of the DNA-RNA hybrid and/or local nucleotide concentrations. Regardless of the mechanism of lesion bypass, combining our estimates of lesion bypass frequency with the lack of fidelity of RNAP over alkyl adducts (21), suggests that alkylating agents can induce considerable transcriptional mutagenesis.

Following completion of TCR, it has been widely thought that RNAP restarts transcription from the site of damage (10). However, recent work on bulky UV-induced cyclobutane pyrimidine dimers (11) challenges the universality of this model, reporting that RNAP dissociates from DNA at the damaged site and subsequent transcription initiation at the genic promoter is required for transcript synthesis. Our results corroborate these latter findings and extend them to the alkylation damage induced by DEN. The observed 5′ bias of repair coupled with mathematical modeling indicates that RNAP does not always restart following repair. Furthermore, through analyzing parameter regimes within bootstrap uncertainty (Fig. 4*C*) and model selection analysis (Fig. 4*D*), we conclude that our data are entirely consistent with RNAP always disassociating after repair. The 5′ repair bias echoes the enhanced 5′ repair found in the damage and repair maps generated from pyrimidine dimers (24) and agrees with the finding that TCR efficiency corresponds to gene length (26). Our finding that transcription does not consistently restart from the stall site following repair is particularly relevant when multiple lesions exist per gene, suggesting that damage-induced expression repression will disproportionately affect long (27), and lowly expressed genes. Supporting this hypothesis, in vitro damage experiments show that the degree of expression reduction was correlated with gene length following exposure to UV, the chemotherapeutic cisplatin, and the cigarette smoke component benzo(a)pyrene (28).

The gradient of mutation density we observe through gene bodies has implications for the accurate modeling of mutation patterns (29, 30), necessary for the prediction of oncogenic selection (31). Our model provides sufficient damage for this gradient to manifest, arising due to inefficient repair at downstream positions caused by the dissociation of RNAP. The codependency of damage burden and expression level enriches the developing mechanistic understanding of mutation patterns over the genome (29, 32). Mutation patterns resulting from a high damage burden are not simply an amplification of the patterns expected from a lower dose of damage.

Quantitatively mapping the consequences of endogenous and exogenous DNA damage is necessary to understand mutagenesis, gene expression dysregulation, and the impact of environmental and therapeutic agents. Here, we have developed an integrative view of TCR following alkyl damage, complementing existing experimental assays that measure individual aspects of this fundamental repair process. Our results exemplify how mechanistic quantitative modeling can be used to bridge the molecular processes of damage and repair through to their presentation in large-scale genomics data.

## Methods

### DNA Sequencing Variant Calling.

The C3H/HeJ mouse strain reference genome assembly C3H_HeJ_v1 (33) was used for read mapping, annotation, and analysis. Mutation calling and quality filtering was performed using whole genome sequencing of 371 DEN-induced liver tumors from n = 104 male C3H mice, as previously reported (16). A minimum variant allele frequency (VAF) threshold of 10% was applied to remove mutation calls from contaminating non-clonal cells. All mutation data were derived from sequence data in the European Nucleotide Archive (ENA) under accession PRJEB37808 and processed files directly used as input for this work are publicly available https://doi.org/10.1038/s41586-020-2435-1. Gene annotation in C3H_HeJ_v1 coordinates was obtained from Ensembl v.91 (34).

### Mutation Phasing.

Genomic segmentation on mutational asymmetry was performed as previously reported (16). In brief, mutational strand asymmetry was scored for each genomic segment using the relative difference metric S = (F−R)/(F+R) where F is the rate of mutations from T on the forward (plus) strand of the reference genome and R the rate of mutations from T on the minus strand (mutations from A on the plus strand). The phasing of mutation asymmetry is agnostic to which base harbors the mutagenic lesion, orthogonal data is required to resolve which asymmetry indicates the lesion containing strand. In the case of A versus T asymmetry from DEN damage, prior studies have established T rather than A modification as the principal mutagenic lesion (16, 35, 36). A mutational asymmetry score of S > 0.33 was used to identify the inheritance of forward strand lesions and S < -0.33 as the inheritance of reverse strand lesions. Analyses were confined to n = 237, clonally distinct DEN induced tumors that met the combined criteria of: i) not labeled as mutationally symmetric (see ref. 17), ii) tumor cellularity >50%, and iii) >80% of substitution mutations attributed to the DEN1 signature (16) by sigFit (v.2.0) (37).

Relative to the reference genome sequence, a plus (P) strand gene is transcribed using the reverse (R) strand as a template. So a P strand gene in a genomic segment with R strand lesions (denoted RP orientation) is expected to be subject to transcription-coupled repair. A minus strand (M) gene with forward (F) strand lesions (FM orientation) is also expected to be subject to transcription-coupled repair, as the retained lesions are on the transcription template strand. Conversely, FP and RM orientation combinations will have lesions on the nontemplate strand for transcription and are therefore not expected to be subject to transcription-coupled repair.

### Gene Expression.

Paired-end, stranded total RNA-seq from C3H male mouse livers not exposed to DEN (n = 4, matching the developmental time of mutagenesis, postnatal day 15, P15) was previously generated and is available from Array Express under accession E-MTAB-8518. RNA-seq was aligned to the reference genome C3H_HeJ_v1 using the splice-aware aligner Star (v2.7.6a). A C3H liver-specific splice junction database was generated from an initial round of RNA-seq read alignment to the C3H_HeJJ_v1 reference genome guided by Ensembl (v.91) genomic annotation. Using the sex-, strain-, and tissue-matched splice junction database, a second iteration of Star alignment produced a final RNA to genome alignment with output attribute flags set to preserve read orientation information (outSAMattributes: NH HI AS nM). The transcription strand of RNA-seq reads was resolved using read-end and mapping orientation extracted by Samtools view (v.1.7.0) and read-pairs exclusively mapping within annotated exons were identified using Bedtools intersect (v.2.29.2). Intronic read-pairs were defined as those mapping within a genic span, derived from a sense-strand transcript, and not in the exonic set. Only read-pairs with a mapping quality (MAPQ) >10 were used to quantify gene expression. Nascent transcription was quantified by counting read-pairs in the intronic set using Bedtools multicov (v.2.29.2). The read count was normalized to reads per kilobase of analyzed intron for each gene in each sequence library and then normalized to nascent transcripts per million (nTPM) for each library. The final nascent transcript expression estimate per gene was taken as the mean of nascent TPM over replicate libraries. Nascent transcription estimates could be generated for 85% (n = 17,304) of protein-coding genes. Overlapping genes, defined by primary transcript coordinates, were hierarchically excluded from analysis: Starting with the most expressed gene, any overlapping less-expressed genes were excluded. Code for this analysis is available at https://github.com/CraigJAnderson/lce-si_nascent.

Genes with similar estimates of nascent expression were aggregated for analysis of transcription-coupled repair. The sigmoidal distribution relating nascent transcription rate to mutation rate (Fig. 2*B*) was segmented using linear regression models in the R package Segmented (v.1.3-3) (38). This defined n = 4,649 genes with zero or low detected nascent expression (<0.287 nTPM) in which reduced mutation rates associated with transcription-coupled repair are essentially undetectable; subsequently stratum 1 genes (light blue in plots). Genes expressed at a greater rate than segmentation threshold >3.73 nTPM do not show a further decrease in mutation rate with increased expression; these n = 7,176 highly expressed genes were defined as stratum 6 (bright red in plots). The n = 4,005 genes with intermediate expression (0.287 to 3.73 nTPM) exhibited a log–linear relationship between expression and mutation rate. These were quantile split into strata 2 to 5, containing approximately 1,000 genes each. The median nascent expression for the six expression strata, in units of nTPM, were 0, 0.49, 1.16, 2.07, 3.14, 11.15.

### Mutation Rates.

Strand resolved mutation rates were calculated as previously described (16, 17). Vectors of 192 categories representing every possible single-nucleotide substitution conditioned on the identity of both the upstream and downstream nucleotides. Each rate being the observed count of a mutation category divided by the count of the trinucleotide context in the analyzed sequence. To report a single aggregate mutation rate, the three rates for each trinucleotide context were summed to give a 64-category vector and the weighted mean of that vector reported as the mutation rate. The vector of weights being the fraction of each trinucleotide in a reference sequence, for example, the composition of the whole genome. Strand-specific mutation rates were calculated with respect to the lesion containing strand, with both mutation calls and sequence composition reverse complemented for reverse strand lesions. Autosomal chromosomes were considered diploid and the X chromosome haploid (all mice were male) for the purposes of calculating mutation rates and sequence composition.

### Mutation Rate versus Expression.

For those genes with measured nascent expression, genes with mean nTPM <0.01 were grouped (n = 1,757), as were genes with mean nTPM>100 (n = 587). The remaining genes were equally split among 15 bins, resulting in a total of 17 expression bins. For each tumor, for each expression bin, the mutation rate due to template strand and nontemplate strand lesions was calculated as detailed above (proportion of mutated bases for given trinucleotide context). The average mutation rate for each strand was calculated similarly but without grouping genes by expression. Observed:expected as a function of expression (Fig. 2 *B*, *Lower*) was calculated as the ratio of template strand mutation rate to the nontemplate strand mutation rate. For each tumor, the expression-dependent observed:expected was fit to a four-parameter log–logistic model using the R package drc (20) (*SI Appendix*, *Extended Data* Fig. S1 *A* and *B*).

### Modeling Transcription-Coupled Repair.

We defined a probabilistic model of lesion detection by RNAP (variable parameter *Pd*), and its subsequent reinitiation (*Pr*) or disassociation (1-*Pr*). The model also incorporated variables for the fraction of lesions that are visible to TCR (*Pv*) and a multiplier parameter (*m*) to translate experimental measurements of nascent TPM (nTPM) to the number of transcription initiations between mutagenesis and DNA replication. The model is illustrated in Fig. 3*A*, and a detailed description is given in *SI Appendix*, *Supplementary File 1*. The model was analyzed both by stochastic simulations (details below) and analytic methods (details in *SI Appendix*, *Supplementary File 1*). The analytic methods were used for parameter inference, which were assessed by simulation. The experimental nascent expression values determined for each stratum (see “*Gene Expression*,” above) were used both for simulated data and for analysis of the tumor data.

### Simulated Mutagenesis and Transcription-Coupled Repair.

For a given parameter set (*Pd*, *Pr*, *m,* *Pv*), we simulated damage and TCR on 1,940,237 phaseable genes, which is the cumulative number of phaseable genes from the mouse liver experiment. For each phaseable gene, the gene length was sampled from the length distribution of the filtered C3H gene list (see above, *Gene Expression*). The gene length was multiplied by the median per base mutation rate [13 × 10^−6^/bp (16)] resulting in the expected lesion number for that gene. The realized lesion number was obtained by sampling a Poisson distribution with mean given by the expected lesion number. Each lesion was placed on the gene at a location determined by sampling from a uniform distribution over [0, gene length]. Each gene was assigned to 1 of 6 expression strata with probabilities given by the strata proportions in the murine data. Each stratum is associated with a measured nascent transcription value *e*, and of the genes in a given stratum we assume a proportion *c* have floor(*e*.*m*) RNAPs that initiate transcription, while the other 1-*c* fraction of genes have floor(*e*.*m*) +1 RNAPs that initiate transcription. For given (*m, e*), *c* is uniquely given by 1-(*e*.*m* - floor(*e*.*m*)) ( *SI Appendix*, *Supplementary File 1*). Thus, for our simulated gene in stratum *e*, we assign either floor(*e*.*m*) or floor(*e*.*m*) +1 RNAPs to initiation transcription with probabilities (*c*, 1-*c*). The RNAPs sequentially initiate transcription, and lesion detection and restart of the polymerases follow the rules illustrated in Fig. 3*A*, potentially resulting in lesion clearance. After all RNAPs have initiated and terminated transcription (potentially before the TES in the case of non-restart), the remaining lesion locations were recorded.

Lesion locations were converted to their position in units of “expected upstream lesions” (base-pair location times 13 × 10^−6^) and a spatial grid of 40 windows of width 0.1 expected lesions was applied (only few genes are long enough for >4 expected upstream lesions, thus further spatial grids would harbor substantial noise). Aggregating over all simulated genes, the summed number of lesions with positions within each spatial window was determined, resulting in the “observed” lesion count. In the absence of TCR, for a given spatial bin, the aggregated lesion number is 0.1 multiplied by the number of phaseable genes with upstream lesion length not exceeding the right boundary of the spatial bin, resulting in the “expected” lesion count for that bin. For each bin, the ratio of the “observed” to the “expected” resulted in the simulated observed:expected mutation rates.

### Parameter Inference on Simulated or Murine Liver Tumor Data.

With input as observed:expected mutation rates with 6 expression strata and 40 spatial windows through the gene in units of expected upstream lesions, parameter inference was performed as follows. Using the numerical output from the obs:exp_theory_ expressions, the Manhattan distance (*L*_1_ norm) between those 6 × 40 measures and the equivalent input data was minimized. Parameter space was initially explored as a grid search. Probabilities *Pd*, *Pr*, and *Pv* were bounded at min = 0, max = 1 with steps of 0.01.

For both simulation and fitting of real data, the parameter range for the expression multiplier m was bounded at min=0.25, max=10 with steps of 0.25. This range was defined following initial grid search exploration with m = 50/i for i = 1, …, 200, the rationale for the parameter bounds is given below in the paragraph “*Plausible Expression Multiplier Parameter Ranges*.” The optimal parameters obtained from the grid search were provided as the starting point for optimization implemented in the R optim function (39) with default parameters to return the final optimized parameter values.

To calculate CIs, the observed:expected mutation rates for the six expression strata were recalculated from the bootstrap sampling of genes (sampling with replacement to original gene list size, n = 1,000 replicates for murine data, n = 100 for simulated data). The inference procedure outlined above was performed for each bootstrapped dataset and reported 95% CIs were calculated as the 0.025 and 0.975 quantiles of bootstrapped parameter estimates.

For AIC-based model selection on the murine data, the measured obs:exp values were assumed to be drawn from a normal distribution with mean obs:exp_theory_ computed as detailed in *SI Appendix*, *Supplementary File 1*, with a common variance *v*. Optimal fits were found by maximizing the likelihood using the “L-BFGS-B” method using the mle2 function from the R package bbmle2 (40). Maximum likelihood estimates for parameters allowing restart were *Pd* = 0.42, *Pr* = 0.66, *m* = 1.59, *Pv* = 0.8, and *v* = 8.8 ∗ 10^−4^; maximum likelihood estimates for parameters without restart were *Pd* = 0.18, *Pr* = 0, *m* = 4.14, *Pv* = 0.8, and *v* = 8.9 ∗ 10^−4^.

### Interpretation of Expression Multiplier *m*.

We assume that, for each expression stratum *k*, for each gene in that stratum, the average number of transcription initiation events between damage and replication, *n*_*k*_, is related to the average expression (nTPM) over all genes in that stratum, *e*_*k*_, by

$$n_{k}=m*e_{k}.$$

The variable *m* can be viewed solely as part of our statistical model; however, it can be given a biological interpretation under some assumptions. Let the number of genes in stratum *k* be *g_k_*. We assume that the gene expression for a given stratum is constant over time and that the RNA sequencing is reflective of this stable expression in the mutagenized cell. If RNA pol II can fail to restart transcription after repair (*Pr* < 1), then not every transcription initiation will result in a transcript; hence, let *s*_k_ be the probability a transcription initiation of a stratum *k* gene results in a transcript. Further, assume that a proportion *p*_k_ of these transcripts are detected in the RNA sequencing. Then, the number of transcripts from stratum *k* detected in the RNA seq would be *g_k_ ∗ n*_k_ ∗ *s*_k_ ∗ *p*_k_.

Recall that by using units of nTPM, the interpretation of the expression level is that for every million nascent transcripts measured, *e*_k_ transcripts are apportioned to each gene in stratum *k*. Therefore, a total of *g_k_ ∗ e_k_* transcripts would be apportioned to stratum *k* for every million transcripts.

Hence,

$$g_{k}*e_{k}=10^{6}*g_{k}*n_{k}*s_{k}*p_{k}/\sum_{k=1}^{6}g_{k}*n_{k}*s_{k}*p_{k},$$

where the right hand side of the equation arises from multiplying 1 million with the proportion of transcripts produced and detected from stratum *k* genes.

So, as by definition *n*_*k*_ = *m* ∗ *e*_*k*_,

$$m=\sum_{k=1}^{6}\left(g_{k}*n_{k}*s_{k}*p_{k}\right)/\left(10^{6}*s_{k}*p_{k}\right).$$

Assuming that the *s*_*k*_ and *p*_*k*_ remain constant over each stratum,

$$m=10^{-6}\sum_{k=1}^{6}g_{k}*n_{k}.$$

Hence, *m* is the number of transcription initiation events (measured in units of million initiations) between damage and replication.

### Plausible Expression Multiplier *m* Parameter Ranges.

We draw on prior literature for plausible parameter values for *m*, which, as discussed above, is the number of transcription initiations (×10^6^) in a cell between DNA damage and replication. Note that when modeling the DEN mutagenesis murine experiment, the number of transcription initiations may not be directly equal to the number of transcripts produced as polymerases may not restart after lesion detection (in the most extreme case with *Pd* = *1, Pr* = *0* and *i* initial lesions, then the number of transcripts produced is equal to the transcription initiations − *i*). However, when comparing to nonmutagenesis experiments, where lesion numbers are expected to be greatly reduced, we equate transcript number and the number of transcription initiations.

For a lower bound on *m*, the number of transcription initiations (×10^6^) between damage and replication, we note that an average time of 2,280 min between damage and DNA replication was estimated from the cell-cycle times of DEN mutagenized rat hepatocytes (41). As the median mRNA half-life has been estimated as 139 min (42), the transcript number measured at any moment can serve as a lower bound for the transcript initiation number; as the typical range estimated is 200,000 to 300,000 transcripts per mammalian cell (43–45), we adopt a lower bound of *m* = 0.25. For a generous upper bound on *m*, we assume: 180,000 chromatin-associated RNA Pol II complexes exist per cell (46); all polymerases are continuously actively transcribing and only transcribing annotated genes; an average transcription rate of 2 kb min^−1^ in mouse liver (22); a median gene length of 60 kb; and again 2,280 min between damage and replication. This implies that 13.68 million transcripts are produced; hence, *m* = 13.68, and thus, *m* = 50 is a further upper bound for the parameter space used in inference. For a reduced upper bound, we note that of the 180,000 chromatin-associated RNA Pol II complexes per cell measured in Kimura et al., only 110,000 were of the hyperphosphorylated form IIO—implying active elongation. Assuming only 110,000 RNA Pol II complexes actively transcribe between damage and replication implies that 8.36 million transcripts are produced; for this reason, our simulated datasets were generated over a grid with an upper bound of *m* = 8.5.

### Orthogonal Estimate of Expression Multiplier *m*.

Bahar Halpern et al. (22) estimated the transcription rate and proportion of time a gene is being transcribed in mouse hepatocytes using single molecule transcript counting; we focus on their periportal samples from mice in the “fed” condition. Taking the product of the estimated transcription parameters, and multiplying by the time between damage and replication (again assumed to be 2,280 min), provides an estimate for the number of transcripts produced by these genes before replication, a per gene estimate of *n*. Seven genes were both measured by single molecule transcript counting (22) and quantified as nTPM from our RNA-seq data. Throughout we have assumed that for each set of genes that are associated to an expression stratum *k*, that *n*_*k*_ = *m* ∗ *e*_*k*_. If now, we assume this holds on a per-gene basis, that is for each gene *n* = *m ∗ e*, then as both *n* and *e* are estimated per gene, we can readily infer *m*. The optimal least square fit for log_10_(*n*) = log_10_(*e*) + log_10_(*m*) resulted in an *m* estimate of 2.77 (*SI Appendix*, *Extended Data* Fig. S2*A*). Note that as the experiments of Bahar Halpern et al. occurred outside of a mutagenesis setting, we have again equated the number of transcripts with the number of transcription initiations *n*.

## Supplementary Material

Appendix 01 (PDF)

## Data Availability

Previously published data were used for this work (16).
