# Supplementary material for: DNA lesion bypass and the stochastic dynamics of transcription-coupled repair
Source: Proc Natl Acad Sci U S A. 2024 May 8;121(20):e2403871121. doi: 10.1073/pnas.2403871121 (PMC11098089; doi:10.1073/pnas.2403871121)
Supplement: Supplementary file 1 — Appendix 01 (PDF) [file pnas.2403871121.sapp.pdf]

Supporting Information for

DNA lesion bypass and the stochastic dynamics of transcription coupled repair

Michael D. Nicholson[1,*], Craig J. Anderson[2], Duncan T. Odom[3,4], Sarah J. Aitken[4,5,6] & Martin S. Taylor[2,*]

[1] CRUK Scotland Centre, Institute of Genetics and Cancer, University of Edinburgh, UK. EH4 2XU

[2] Institute of Genetics and Cancer, University of Edinburgh, Edinburgh, UK. EH4 2XU

[3] German Cancer Research Center (DKFZ), Heidelberg, Germany

[4] Cancer Research UK Cambridge Institute, University of Cambridge, Cambridge, UK

[5] Medical Research Council Toxicology Unit, University of Cambridge, Cambridge, CB2 1QR, UK

[6] Department of Histopathology, Cambridge University Hospitals NHS Foundation Trust, Cambridge, CB2 0QQ, UK

* Correspondence to Michael D. Nicholson or Martin S. Taylor.

Email: michael.nicholson@ed.ac.uk or martin.taylor@ed.ac.uk

This PDF file includes:

Extended Data Figures 1 & 2
Supplementary File 1: Mathematical model for DNA damage and transcription coupled repair

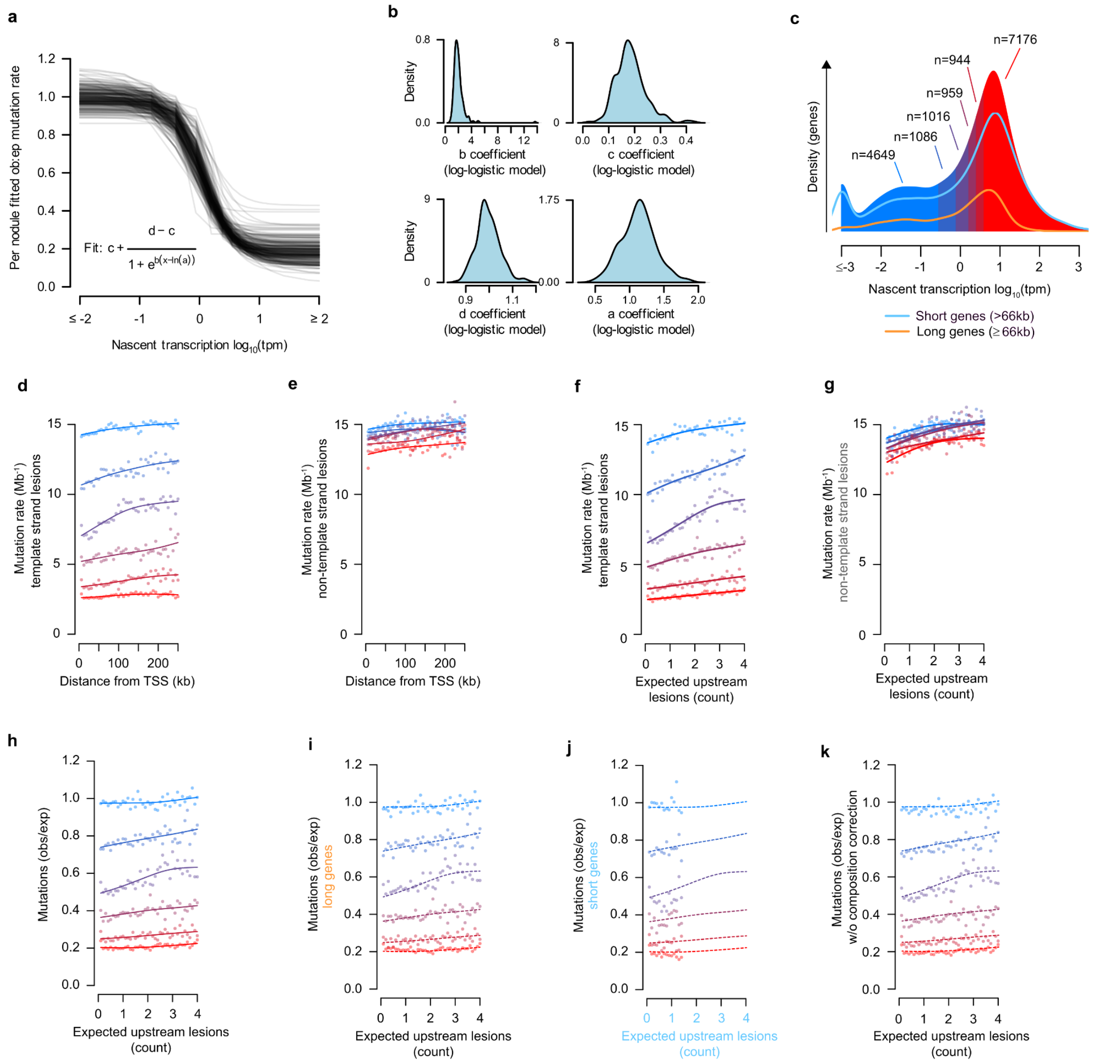

Extended Data Figure 1 | a, Per nodule observed:expected values versus nascent transcription fitted with log-logistic function. **b,** Density plots of fitted coefficients from best-fit log-logistic functions. Coefficient *b* reflects decay rate; Coefficient *c* reflects y-axis value for x → +∞; Coefficient *d* reflects y-axis value for x → -∞; Coefficient *a* reflects inflection point. Unimodality of density plots indicates consistency of effects of TCR across individual nodules. **c,** Distribution of genes by measure of nascent gene expression. Blue→red denote increasing categories of nascent gene expression (thresholds as per Fig 2.b). Density shown for all genes included in gene-body analysis (n=15,830) and scaled density curves shown for the long-gene (≥66 kb, orange) and short-gene (<66 kb, blue) component subsets. **d,** Mutation rates for genes with template strand lesions. Genes classified by expression strata and mutation rates calculated in 5 kb consecutive windows from the TSS. Curves show best-fit splines with 3 degrees of freedom (also shown in Fig 2.c). **e,** As for d but considering genes with non-template lesions (also shown in Fig 2.d). **f,** Analysis as in panel d but distance from TSS

converted to expected upstream lesion count (x-axis) by per-tumour normalisation (Fig 2.e). **g,** as for f but showing genes with non-template lesions. **h-k,** Observed versus expected mutations for each expression strata, calculated as the ratio of template strand (panel f) to non-template (panel g) strand mutations, after adjusting for expected upstream lesions. Lower values indicate greater transcription coupled repair. All genes (**h**; also shown in Fig 2.f), long-genes only (**i**), short-genes only (**j**); all genes without applying mutation rate corrections for sequence composition (**k**); dashed curves show the best-fit splines from panel h for comparison.

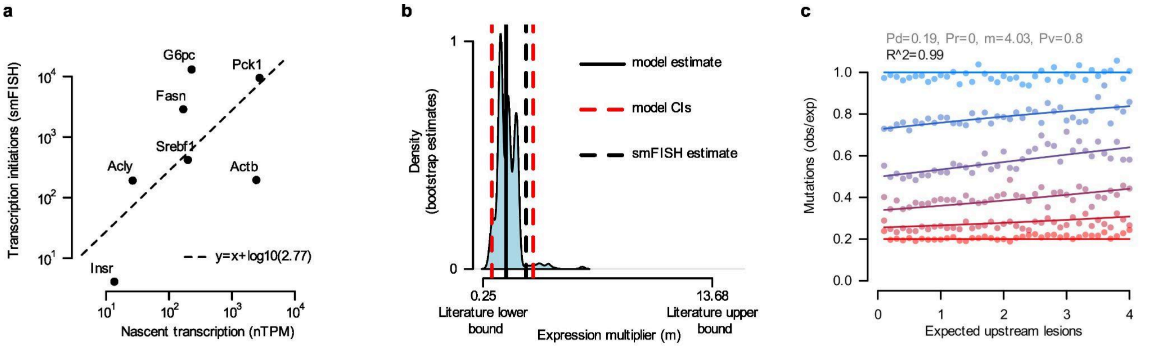

Extended Data Figure 2 | a, Combining single-molecular fluorescence *in situ* hybridisation (smFISH) (Bahar Halpern et al. 2015) with the estimated time between damage and replication, gives an estimate of the transcription initiation number per gene (y-axis) (Methods), for the 7 genes with both smFISH and nascent transcription (nTPM, x-axis) measures. Via the assumed relation, *n*=*m***e*, the least squares fit (dashed-line) provides an estimate of *m*=2.77. **b,** Density of expression multiplier *m* estimates obtained from fitting the mathematical model to bootstrapped data, with x-axis limits displaying plausible parameter range from prior literature (Methods). Dashed black line displays the *m* estimate from **a** which falls within the bootstrap confidence intervals. **c,** Optimal fit of the mathematical model to the murine liver tumour data assuming RNAP never restarts from the damage site following TCR.

DNA lesion bypass and the stochastic dynamics of transcription coupled repair

Supplementary File 1: Mathematical model for DNA damage and transcription coupled repair

Document overview

This document provides the mathematical details for the analytic model of transcription coupled repair.

Method overview

Our aim is to establish a mathematical model, linking fundamental parameters of TCR, to the aggregate mutational pattern seen over genes and individual tumours.

As for a given gene the mutation pattern may be influenced by genic sequence composition and individual tumour mutational burden, we compared observed:expected mutation rates normalised to the expected number of upstream lesions, see Fig 2.e in the main text. Using the 'gene distance' variable of expected number of upstream lesions, all genes can be treated identically - except for gene length (in units of expected upstream lesions) and expression (modelled as the number of RNAPs that initiate transcription prior to the first cell division of the original mutagenised cell). Hence, we consider how the mutational profile for a given gene alters as transcription coupled repair proceeds; the aggregrate profile expected to be seen over all tumours, for all genes of a given length and having seen n RNAPs initiate transcription, may then be acquired for a given set of model parameters by summing over the single gene mutational profie.

Model and methods strategy

We take a gene body of length l which may have lesions placed upon it. We assume the number of lesions up to a position x in the gene is distributed as Pois(μx), where μ is the initial lesion rate; we keep this in the model for generality however as we measure gene position in units of 'expected upstream mutations', $\mu = 1$ when we compare to the data. We assume n RNA polymerases begin transcription at the TSS. These polymerases are ordered (the first RNAP starts transcription then the second etc). If a polymerase encounters a lesion, the lesion is detected with probability p_d. If detected, it is repaired. After repair the polymerase may restart with probability p_r, if not it falls from the strand. We also assume that a certain proportion of lesions are invisible to the RNA polymerases; each lesion is visible with probability p_v. We are interested in the number of remaining lesions after n RNA polymerases have exited the gene. See Fig 4 in the main text for a graphically summary of the mode.

Our aim is to determine the expected number of lesions that remain after n RNA polymerases have exited the gene (either via disassociation after repair or by reaching the transcription end site), in any increment of the gene body (e.g. $[x_1, x_2] \subset [0, l]$). To do so, we pick a position x in the gene and then investigate the probability we have j lesions remaining after n RNA polymerases have exited the gene given that we start with i lesions before x. This amounts to deriving the transition probabilities in a specific Markov chain. From the transition probabilities, we can obtain the expected number of remaining lesions after n polymerases, given we start with i initial lesions. As the number of initial lesions before x is a Poisson random variable, we then average over the initial lesion number to obtain the mean remaining lesions before x. From the mean we can deduce the expected number of lesions remaining in any increment in the gene, and over multiple

genes. The expected number of lesions with repair (template strand), compared to the expected number of lesions in the absence of any repair (non-template strand) is comparable to the experimental data, and is given by Equation 7 below. The derivation of Equation 7, and how to numerically evaluate the expression is the purpose of this document.

Probability transition matrix

We take a position x in the gene, and suppose we have i initial lesions on the template strand before x. For now assume all lesions are visible, that is $p_v = 1$. We now sequentially send through polymerases and let L_n denote the number of remaining lesions after n polymerases have exited the gene, so here $L_0 = i$. We seek $\mathbb{P}(L_n = j|L_0 = i)$. As L_n is a Markov chain, it is sufficient to consider $\mathbb{P}(L_1 = j|L_0 = i)$, from which $\mathbb{P}(L_n = j|L_0 = i)$ can be obtained by appropriate matrix multiplication according to standard Markov chain theory. We turn to $\mathbb{P}(L_1 = j|L_0 = i)$.

The strategy is to first mark each of the i initial lesions depending on whether the polymerase will detect these, i.e. we binomially thin the lesions, and then consider how many of the thinned lesions the polymerase will detect before exiting the increment of interest (falling off the gene or passing beyond the position x).

Let L'_0 be the number of lesions the RNAP will detect if encountered before x, so L'_0 is binomial with i trials and with success probability p_d. Then the number of lesions repaired by the first polymerase, R_1 is geometric with parameter $q_r = 1 - p_r$ truncated to be within $1, \dots, L'_0$. So for $k = 1, \dots, L'_0$

$$\mathbb{P}(R_1 = k) = \begin{cases} p_r^{k-1} q_r, & k < L'_0 \\ p_r^{L'_0 - 1}, & k = L'_0 \end{cases}$$

If $L'_0 = 0$, then $\mathbb{P}(R_1 = 0) = 1$.

If the remaining lesion number after the first RNAP is $L_1 = j$, then $i - j$ lesions have been repaired, so

$$\mathbb{P}(L_1 = j|L_0 = i) = \mathbb{P}(R_1 = i - j) = \sum_{y=0}^{i} \mathbb{P}(R_1 = i - j|L'_0 = y)\mathbb{P}(L'_0 = y). \tag{1}$$

We introduce the indicator $1(A)$ which returns 1 when the condition A is true and 0 otherwise. Notice that no lesions are repaired only if all lesions are undetected, that is $\mathbb{P}(L_1 = i|L_0 = i) = \mathbb{P}(L'_0 = 0)$. Hence separating out this case, and using that $j \leq i$,

$$\begin{aligned} \mathbb{P}(L_1 = j|L_0 = i) &= 1(j = i)\mathbb{P}(L'_0 = 0) + 1(j < i) \sum_{y=1}^{i} \mathbb{P}(R_1 = i - j|L'_0 = y)\mathbb{P}(L'_0 = y) \\ &= 1(j = i)\mathbb{P}(L'_0 = 0) \\ &\quad + 1(j < i) \sum_{y=1}^{i} \left[p_r^{i-j-1} q_r 1(i - j < y) + p_r^{y-1} 1(i - j = y) \right] \binom{i}{y} p_d^y q_d^{i-y}. \end{aligned}$$

In the last step above, within the summation, we separated the cases where the RNAP falls off before encountering all damage that would be detected $(1(i - j < y))$ and all damage that would be detected is encountered $(1(i - j = y))$. We can extend this to $\mathbb{P}(L_1 = j|L_0 = i)$ for $j > i$ by setting the probability to 0 in this case.

Let P be the matrix with elements $P_{i,j} = \mathbb{P}(L_1 = j|L_0 = i)$, and $P^{(k)}$ be P multiplied by itself k times. Then from standard Markov chain theory we identify

$$\mathbb{P}(L_n = j|L_0 = i) = P_{i,j}^{(n)}.$$

If L_0 is known then P would be of size $L_0 \times L_0$ as the number of lesions before x can take values in $\{0, \dots, L_0\}$. However, as we assume L_0 is Poisson, then L_0 is in principle unbounded. Momentarily, to be justified shortly below, assume $L_0 \leq L_{\max}$ for some integer $L_{\max}$. Then P would be of size $L_{\max} \times L_{\max}$.

Expected number of lesions remaining

We again concern ourselves with the lesions remaining before x. From the above, with fixed L_0, we can obtain $\mathbb{P}(L_n = j|L_0)$, and thus determine the expected number of lesions remaining

$$\mathbb{E}[L_n|L_0] = \sum_{j=0}^{L_0} \mathbb{P}(L_n = j|L_0)j.$$

Note $L_n \leq L_0$, justifying the summation limits. Our primary aim is

$$\mathbb{E}[L_n] = \sum_{i=0}^{\infty} \mathbb{E}[L_n|L_0 = i]\mathbb{P}(L_0 = i) \tag{2}$$

with L_0 distributed as a Poisson variable with mean μx. Equation (2) contains an infinite sum, however for some precision parameter $\delta > 0$, we may choose L^{δ}_{max} such that

$$\mathbb{E}[L_n] - \sum_{i=0}^{L^{\delta}_{\text{max}}} \mathbb{E}[L_n|L_0 = i]\mathbb{P}(L_0 = i) < \delta,$$

due to the following argument.

First notice that the number of lesions is monotone decreasing in n. Hence for any n, $\mathbb{E}[L_n|L_0 = i] \leq i$, and so,

$$\begin{aligned}
\mathbb{E}[L_n] - \mathbb{E}[L_n 1(L_n \leq L^{\delta}_{\text{max}})] &= \sum_{i=L^{\delta}_{\text{max}}+1}^{\infty} \mathbb{E}[L_n|L_0 = i)\mathbb{P}(L_0 = i) \\
&\leq \sum_{i=L^{\delta}_{\text{max}}+1}^{\infty} i\mathbb{P}(L_0 = i) \\
&= \mathbb{E}[L_0] - \sum_{i=0}^{L^{\delta}_{\text{max}}} i\mathbb{P}(L_0 = i).
\end{aligned}$$

Thus if we wish to compute $\mathbb{E}[L_n]$ with an error of at most $\delta > 0$, we choose L^{δ}_{max} such that

$$\mu x - \sum_{i=0}^{L^{\delta}_{\text{max}}} i\mathbb{P}(L_0 = i) \leq \delta,$$

which may be found numerically. If we wish to evaluate $\mathbb{E}[L_n]$ at a grid of positions x_1, $x_2 \ldots, x_y$, then we can carry out the procedure above for each x_i, obtaining a sequence of L^{δ}_{max} and then select the maximum of these. When comparing with the experimental data, we selected $\delta = 10^{-5}$, and work with

$$\sum_{i=0}^{L^{\delta=10^{-5}}_{\text{max}}} \mathbb{E}[L_n|L_0 = i]\mathbb{P}(L_0 = i).$$

Until now we have assumed all lesions are visible. However if $p_v < 1$, then by Poisson thinning the arguments presented above holds identically but with

$$\mathbb{E}[L_n] \mapsto \mu x(1 - p_v) + \mathbb{E}[L_n],\ L_0 \sim \text{Pois}(\mu x p_v).$$

Observed vs expected lesion density over multiple genes including invisible lesions

If we suppose we have k genes then the expectation of the observed lesion count on the template strand up to position x, summed over the k genes is

$$\mathbb{E}[L^{(k)}_n] = k(\mu x(1 - p_v) + \mathbb{E}[L_n]).$$

In the absence of repair, representing the setting of the non-template strand, the expected number of mutations is

$$k\mu x.$$

Often we work with the density in an increment between two points x_{i-1} and x_i. Here the expectation of the observed number of lesions over k genes in the increment is

$$\begin{aligned}\mathbb{E}[L_n^{(k)}(x_i)] - \mathbb{E}[L_n^{(k)}(x_{i-1})] &= k(\mu x_i(1-p_v) + \mathbb{E}[L_n(x_i)]) - k(\mu x_{i-1}(1-p_v) + \mathbb{E}[L_n(x_{i-1})]) \quad (3)\\ &= k(\mu(1-p_v)(x_i - x_{i-1}) + \mathbb{E}[L_n(x_i)]) - \mathbb{E}[L_n(x_{i-1})]). \quad (4)\end{aligned}$$

The expected number of lesions in the absence of repair in the increment is

$$k\mu(x_i - x_{i-1}).$$

Therefore, the observed over expected number of lesions in the increment is

$$1 - p_v + \frac{\mathbb{E}[L_n(x_i)]) - \mathbb{E}[L_n(x_{i-1})]}{\mu(x_i - x_{i-1})}. \quad (5)$$

Mixed number of polymerases and data fitting

We use Equation (5) as the basis to compare against the experimental data, and for convenience define this as

$$f(i,n) = 1 - p_v + \frac{\mathbb{E}[L_n(x_i)]) - \mathbb{E}[L_n(x_{i-1})]}{\mu(x_i - x_{i-1})}, \quad (6)$$

where i ranges over the position in the gene and n denotes the number of polymerase that have initiated transcription. The number of polymerases that have initiated transcription is unknown. Instead we have measures of nascent transcript levels of the genes under consideration at the time of mutagenesis in untreated mice. As discussed in the main text we bin the genes according to 6 expression strata. Let e_j be the average expression level of genes in strata j (in units of nTPM), and suppose an average of n_j polymerase initiate transcription for strata j genes. To relate these quantities we introduce the expression multiplier m (common over all strata) such that

$$n_j = me_j.$$

Hence $1/m$ is the 'expression contribution' per polymerase (n_j units of $1/m$ is the measured expression). When fitting this model to data we are tightly constrained as the number of polymerases in the model must be an integer. To increase flexibility and realism, for each strata j, we assume a mixed population of genes such that a proportion c_j (with $c_j \in (0,1]$) of genes have integer $n_j^{(1)}$ polymerases initiating transcription and $1 - c_j$ have $n_j^{(1)} + 1$ polymerases. Therefore the observed versus expected number of mutations for strata j would be described by

$$\text{obs:exp}_{\text{theory}} = c_j f(i, n_j^{(1)}) + (1-c_j) f(i, n_j^{(1)} + 1) \quad (7)$$

Keeping the interpretation that $1/m$ is the expression contribution per polymerase, then in this setting the measured expression can be written as

$$e_j = m^{-1} c_j n_j^{(1)} + m^{-1}(1-c_j)(n_j^{(1)} + 1),$$

and so

$$me_j - n_j^{(1)} = 1 - c_j. \quad (8)$$

Now in addition to the original parameters (p_d, p_r, p_v, m) we have $(c_1, \ldots, c_6)$. However, these additional 6 parameters are constrained due to the following argument: Notice that for each j the triple $(me_j, n_j^{(1)}, c_j)$ is constrained by Equation 8 and that

$$n_j^{(1)} = me_j - (1 - c_j).$$

If me_j is given, then as $1 - c_j \in [0, 1)$, the only integer in the interval $(me_j - 1, me_j]$ is $\text{floor}(me_j)$ and hence the unique choice for $n_j^{(1)}$ is

$$n_j^{(1)} = \text{floor}(me_j). \tag{9}$$

Which immediately implies

$$c_j = 1 - (me_j - \text{floor}(me_j)). \tag{10}$$

Thus our search space has only 4 dimensions. For a given set of (p_d, p_r, p_v, m), the 'lower' integer polymerase numbers $n_j^{(1)}$ is found using Equation 9 followed by the mixing proportions for each strata c_j using Equation 10. Then the observed:expected mutation rate for each strata j over spatial positions i is given by Equation 7 where $f(i, n)$ is defined in Equation 6. The numerical output of Equation 7 is minimised against the experimental data using the l_1 norm to find optimal parameter estimaties for (p_d, p_r, p_v, m).
